# Obituary

**Published:** 1866-06

**Authors:** 


*Obituary.*—The Memphis "Medical and Surgical Monthly" brings us the sad intelligence of the death of Dr. E. D. FENNER, of New Orleans. His loss to society and the medical profession we deplore. He was known to us as an able and useful medical writer, and honorable gentleman. As a teacher he won the confidence and respect of those who knew him in connection with the new "School of Medicine" in New Orleans.

